# On the Selective Packaging of Genomic RNA by HIV-1

**DOI:** 10.3390/v8090246

**Published:** 2016-09-12

**Authors:** Mauricio Comas-Garcia, Sean R. Davis, Alan Rein

**Affiliations:** 1 HIV Dynamics and Replication Program, Center for Cancer Research, National Cancer Institute, Frederick, MD 21702, USA; mauricio.comasgarcia@nih.gov; 2 Genetics Branch, Center for Cancer Research, National Cancer Institute, Bethesda, MD 20892, USA; sdavis2@mail.nih.gov

**Keywords:** retroviruses, retroviral RNA, virus assembly, HIV-1, selective RNA packaging, genomic RNA, packaging, encapsidation, capsid, RNA-protein interactions

## Abstract

Like other retroviruses, human immunodeficiency virus type 1 (HIV-1) selectively packages genomic RNA (gRNA) during virus assembly. However, in the absence of the gRNA, cellular messenger RNAs (mRNAs) are packaged. While the gRNA is selected because of its *cis*-acting packaging signal, the mechanism of this selection is not understood. The affinity of Gag (the viral structural protein) for cellular RNAs at physiological ionic strength is not much higher than that for the gRNA. However, binding to the gRNA is more salt-resistant, implying that it has a higher non-electrostatic component. We have previously studied the spacer 1 (SP1) region of Gag and showed that it can undergo a concentration-dependent conformational transition. We proposed that this transition represents the first step in assembly, i.e., the conversion of Gag to an assembly-ready state. To explain selective packaging of gRNA, we suggest here that binding of Gag to gRNA, with its high non-electrostatic component, triggers this conversion more readily than binding to other RNAs; thus we predict that a Gag–gRNA complex will nucleate particle assembly more efficiently than other Gag–RNA complexes. New data shows that among cellular mRNAs, those with long 3′-untranslated regions (UTR) are selectively packaged. It seems plausible that the 3′-UTR, a stretch of RNA not occupied by ribosomes, offers a favorable binding site for Gag.

## 1. Introduction

Human immunodeficiency virus type 1 (HIV-1), the causative agent of acquired immunodeficiency syndrome (AIDS), is a retrovirus. Retroviruses are assembled from a few thousand copies of a single structural polyprotein, termed “Gag”. The particles contain much smaller numbers of other virus-coded polyproteins and RNA, and are surrounded by a lipid bilayer derived from the plasma membrane of the virus-producing cell. After the viral particle is released from the cell, a series of proteolytic cleavages transform it from the non-infectious “immature” form to the infectious “mature” form; however, this review will only deal with immature particle assembly.

The major domains of the Gag protein are matrix (MA), capsid (CA), nucleocapsid (NC), and p6. There are also two short “spacer” regions, SP1 and SP2, flanking the NC domain. Briefly, the MA domain is involved in the interaction of Gag with membranes, but can also bind to RNA; the CA domain mediates most of the protein-protein interactions between Gag molecules; NC, which is positively charged and contains two small zinc fingers, plays a major role in interactions of Gag with nucleic acids; and p6 contributes to the release of the assembled particle from the host cell by interacting with the cellular endosomal sorting complexes required for transport (ESCRT) machinery.

The genomic RNA (gRNA) of HIV-1 is ~9.7 kb long. All retrovirus particles contain a dimer of gRNA, in which two molecules of the same polarity are joined together by a limited number of base-pairs [1,2]. Packaging of the gRNA is an essential part of the HIV-1 infectious cycle, and yet some key steps of this process remain elusive. Virion assembly occurs at the plasma membrane and is coupled to RNA packaging; however, it is still not clear where and how the gRNA selectively interacts with Gag. Selective packaging of the gRNA requires a *cis*-acting RNA element (called the *packaging signal* or Ψ) that begins in the 5′-untranslated region (UTR) and extends into the Gag open reading frame (ORF) [3,4]. Unfortunately, however, there is still no molecular mechanism that explains how the gRNA is preferentially packaged in the presence of a large excess of cellular and spliced viral RNAs. While it is clear that the zinc fingers within NC are essential for specific packaging of the gRNA [3,5], it is not known whether other domains of Gag are involved. It is interesting to note the suggestion that, in single-stranded RNA icosahedral viruses, selective packaging of the viral RNA results from a delicate balance between the relative strengths of RNA-protein and protein-protein interactions [6]. By analogy, it seems possible that selective packaging of the gRNA in HIV-1 depends on the strength of Gag–Gag, as well as on Gag–RNA interactions.

## 2. The Role of RNA in Particle Assembly

As in most, if not all viruses that package single-stranded RNA, the assembly of HIV-1 virions requires the presence of RNA. The RNA is incorporated during, not after, the process of capsid formation. Thus, the gRNA not only carries genetic information, but also contributes to the assembly of the structural proteins into a virion. However, it is important to realize that this is not a unique property of the gRNA. In the absence of the gRNA, the HIV-1 Gag, as well as the Gag from other retroviruses, assembles in cultured cells into virus-like particles (VLPs) [7]. The amount of RNA in these Ψ-HIV-1 VLPs, as well as the morphology of the particles, is indistinguishable from that of true virions [8]. Similarly, recombinant Gag proteins can assemble into particles in vitro, but this assembly requires the addition of nucleic acids; nearly any single-stranded nucleic acid longer than ~20–30 nucleotides can support assembly under these conditions [9]. This means that, at least in vitro, HIV-1 VLP assembly does not depend on the length of the packaged RNA: proper Gag–Gag interactions can occur even when the RNA is extremely small compared to the length of the gRNA (~9.7 kb). In fact, these observations imply that binding to nucleic acid, even a very short nucleic acid, somehow primes Gag for assembly. How does nucleic acid-binding contribute to assembly?

A very significant clue to this riddle was uncovered by Zhang et al., who reported that if the NC domain of Gag is replaced by a protein that can form dimers (leucine zippers) without binding to nucleic acids, then the resulting chimeric protein assembles well in mammalian cells [10]. These particles are morphologically almost indistinguishable from immature particles formed by wild-type Gag, but contain little, if any, RNA [11]; their structure is presumably maintained entirely by protein-protein interactions.

## 3. Gag–RNA Interactions Involve Multiple Domains of Gag

Although NC may play a predominant role in the interactions of Gag with RNAs, there are many kinds of evidence showing that the MA domain also binds RNAs. Thus, the binding of the MA domain to RNA in reticulocyte lysates apparently prevents the association of Gag with (and assembly at) cytoplasmic membranes [12]. In vitro, the binding of the MA domain to RNAs interferes with the optimal RNA-chaperone activity of Gag [13]. Finally, RNA cross-linking-immunoprecipitation analysis shows that the MA domain of Gag protein within virus-producing cells is bound to RNAs, principally transfer RNAs (tRNAs) [14]. Moreover, when nucleic acid is added to recombinant Gag (lacking the p6 domain), particles assemble in vitro, but these particles are far smaller than authentic virions: indeed, they are so small that they cannot contain the extended, rod-shaped Gag molecules found in authentic immature particles [9]. Particles of the correct size are formed if most of the MA domain is deleted, or if a preferred ligand for the MA domain, such as inositol pentakisphosphate (IP5), is added [15]. These observations are all consistent with the fact that free Gag is very flexible [16,17], and both its MA domain (at its N-terminus) and its NC domain (near its C-terminus) can bind either a lipid bilayer or nucleic acid [18,19]. Gag extends into a rod when both lipids (the preferred ligand of the MA domain) and nucleic acid (the preferred ligand of the NC domain) are present simultaneously [18].

## 4. A Switch in Gag: The Decision to Assemble

Although binding of Gag to RNA is mostly driven by the interactions between the NC domain and the nucleic acid, there is some evidence that Gag–Gag interactions play an important role in stabilizing Gag–RNA complexes. For example, it has been shown that deletion of the C-terminal domain (CTD) of CA (responsible for Gag dimerization in solution) decreases the stability of Gag–RNA complexes, both in the cytoplasm and at the plasma membrane [20]. This deletion also decreases the ability of Gag to associate with the viral RNA in the cytoplasm. Unpublished data from our laboratory indicates that Gag–Gag interactions through the CTD of Gag are key factors in the specificity of Gag–RNA interactions [21]. Taken together, these observations suggest that Gag–RNA interactions not only depend on the strength of Gag–RNA interactions but also on Gag–Gag interactions.

**Figure 1:**
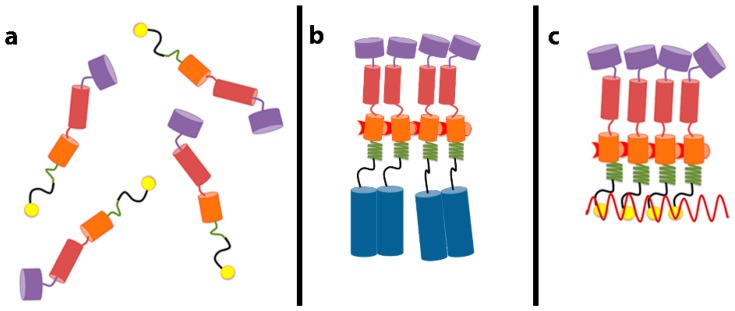
Schematic depiction of changes in Gag leading to assembly. (**a**) Gag contains several domains: matrix (MA, purple), the N-terminal domain of capsid (CA, red), the C-terminal domain of CA (orange), spacer 1 (SP1) (green), nucleocapsid (NC, yellow), and spacer 2 (SP2) and p6 (not shown). SP1 is unstructured in free Gag; (**b**) When NC is replaced by a dimerizing leucine zipper (blue cylinders), the dimerization mediated by the zipper induces SP1 to fold into a helical conformation. We propose that this leads to the appearance of new interfaces within CA for Gag–Gag interaction; (**c**) Gag molecules can also be brought into close proximity by binding cooperatively to an RNA molecule (wavy red line). This also causes helix formation in SP1 and interface formation in CA. We propose that this is a step in normal virion assembly.

As noted above, RNA is unnecessary for particle assembly if the Gag protein contains a dimerizing zipper domain [10,11]. This implies that the role of RNA in assembly is to bring Gag molecules into close proximity: once this happens, the Gag molecules are able to engage in all the protein-protein interactions required for assembly. How does juxtaposition of Gag molecules alter their properties? We have recently identified a small region of Gag whose conformation responds to its local concentration [22]. As mentioned above, the SP1 domain is located between the NC domain (the principal RNA-binding domain) and the CA domain, which performs most of the Gag–Gag interaction in assembly. The sequence of SP1 is such that if it were folded into an α-helix, the helix would be amphipathic, with a polar face and a hydrophobic face. Thus, if multiple SP1 molecules were associated together, they could form bundles of helices, with hydrophobic faces shielded from the aqueous solvent in the interior of the bundles. We have analyzed the properties of short fragments, including SP1, of Gag and of assembly-competent Gag–zipper chimeric proteins [23]. We found that free SP1 peptide is helical when it is at high (~5 mM), but not at low (~0.05 mM) concentration. In contrast, it is helical even in very dilute solutions when it is attached to a dimerizing leucine-zipper, which of course maintains it at a high local concentration [22]. We also noted that these SP1-zipper proteins are tetrameric in solution. The tetramers are formed because the zipper moiety causes them to dimerize, and then the SP1 moiety causes the dimers to further dimerize into tetramers. Mutations in SP1, which disrupt proper assembly in the Gag or Gag–zipper context, also fail to convert the SP1-zipper dimers to tetramers; this shows that the same SP1–SP1 interactions inducing tetramerization of the SP1-zipper constructs are important in proper particle assembly by Gag or Gag–zipper protein. We proposed that juxtaposition of Gag molecules could induce helix formation in the SP1 domain and that this change in SP1 conformation might be propagated into the CA domain, activating the formation of new interfaces for Gag–Gag interaction in assembly. This scenario, presented schematically in Figure 1, can explain how the close proximity of Gag molecules can lead to particle assembly. Three-dimensional structures of this region of Gag in the helical form have now been elucidated [24,25].

## 5. Selective Packaging and Ψ

In general, the packaging signals of retroviruses are located in and near the 5′-UTR of the gRNA and are several hundred bases long. Surprisingly, the Ψ of HIV-1 is still not very well defined; as far as is known, there is no single, contiguous stretch of the gRNA which confers selective packaging upon a heterologous RNA [26]. (In other retroviruses, such stretches have been identified [27,28,29]). However, deletion and mutational analysis shows that sequences near the 5′ end of gRNA are crucial for selective packaging [3,30,31]. There are several plausible scenarios that could conceivably explain Ψ-mediated selective packaging of the HIV-1 gRNA: (i) Ψ is a high-affinity binding site for Gag; hence, in the cell, HIV-1 Gag is preferentially bound to the gRNA; (ii) the binding of Gag to RNA is cooperative, and the binding of Gag to Ψ has a higher degree of cooperativity than to non-Ψ RNAs; (iii) Ψ acts as a substrate that lowers the energetic barrier for virus nucleation such that assembly on gRNA is more efficient than assembly on other RNAs; and (iv) some combination of these scenarios.

Studying how Ψ controls selective packaging of the gRNA in cell culture is extremely challenging; the 5′-UTR controls RNA expression, RNA dimerization, and splicing, and thus deletions or mutations within this region have profound effects on the HIV-1 infectious cycle, other than altering Gag–RNA interactions. This is one of the many reasons that have prompted the study of Gag–RNA interactions in vitro using purified components. Furthermore, analysis of binding in vitro might enable us to determine the binding mechanism and to measure binding energies and rates. Most in vitro studies have focused on testing the hypothesis that Ψ is a high-affinity binding site for Gag; hence, they have concentrated on measuring the affinity of Gag, as well as NC, to RNAs (≤600 bases) that are small compared to the length of the HIV-1 gRNA [32,33,34]. It is technically challenging to measure Gag–RNA affinities, since, as noted above, the interaction of Gag with RNA can lead to particle assembly. However, these measurements can be successfully performed if the Gag and RNA concentrations are low enough that Gag–RNA complexes do not associate with each other into particles.

Another inherent complexity in these measurements arises from the fact that Gag contains positively charged stretches: both the MA and NC domains are polycationic and will therefore have significant affinity for any nucleic acid. In fact, this non-specific RNA binding is not merely a complication for the investigator, but is part of the reason why selective packaging defies a facile explanation: the gRNA in the cell is in the presence of a large excess of other RNAs. In fact, cellular messenger RNAs (mRNAs) are packaged in cells containing Gag but lacking Ψ-containing RNA [8,35]; thus, these RNAs are fully capable of being encapsidated. In other words, they are in competition with gRNA for encapsidation, and Ψ confers an advantage upon gRNA in this competition. Understanding selective packaging will require an understanding of this advantage.

One experimental strategy that has been employed to “block out” the non-specific component of Gag–RNA binding in vitro is to measure binding to the RNA of interest in the presence of an excess of irrelevant competitor RNA, such as tRNA [32,36]. This was done over 20 years ago by Berkowitz et al., who monitored binding to glutathione-*S*-transferase-tagged Gag by electrophoretic mobility shift assay in a >1000-fold excess (by mass) of tRNAs [36]. Under these conditions, Gag binds preferentially to a Ψ-containing RNA rather than a control RNA; thus, it is clear that when non-specific binding is eliminated by the presence of a competitor, Gag has a higher affinity for Ψ than for other RNAs. However, if enough tRNA was added, binding of Gag to the Ψ RNA was also inhibited. In the cell, of course, selective packaging occurs despite the large excess of competing RNAs; thus it seems unlikely that this difference in binding affinity could explain selective packaging.

Another strategy was used by Webb et al. to eliminate non-specific binding [37]. They measured the relative contribution of non-specific and specific interactions in Gag–RNA binding by adding increasing amounts of salt to pre-bound Gag–RNA complexes. Remarkably, they found that Gag–Ψ complexes are far more salt-resistant than complexes containing a control RNA. This striking observation shows that there is a difference in character between the binding of Gag to Ψ and its binding to the control RNA. Specifically, binding of Gag to Ψ has a much stronger non-electrostatic (specific) component than binding to a control RNA. Their results also indicated that Gag binds to Ψ with a lower effective charge than when it binds to a control RNA. Based on these results Webb et al. proposed a binding model in which Gag binds to a Ψ-RNA through the NC domain, while binding to a non-Ψ RNA involves both the MA and NC domains [37]. It is important to note that, while raising the ionic strength of the assay reveals the special character of binding to Ψ, it cannot on its own explain selective packaging: virus assembly occurs under physiological conditions, not in elevated salt concentrations (however, we cannot exclude the theoretical possibility that selective binding occurs in some unique location within the cell where the ionic strength is very high). Taken together, these observations suggest that selective packaging of the HIV-1 gRNA cannot be driven only by a high-affinity Gag–Ψ binding mechanism.

## 6. Packaging Non-Viral RNAs

**Figure 2:**
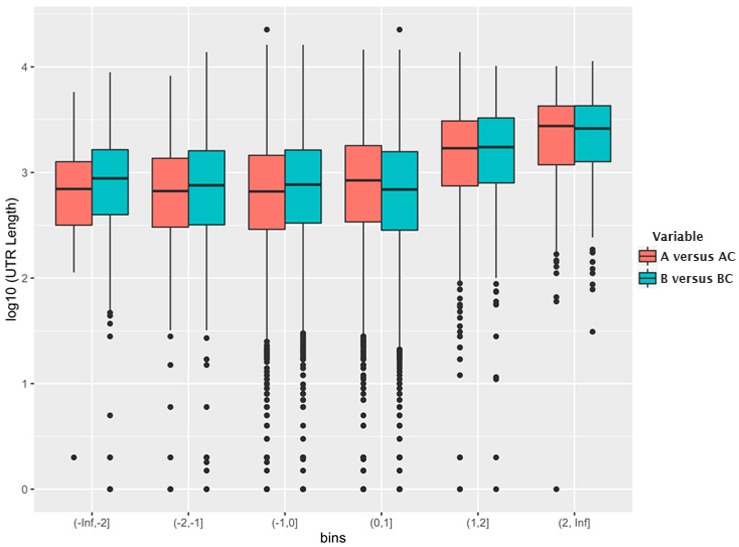
Relationship between bins of similar log_2_ (fold change) on the *x*-axis and the log_10_ (UTR length) on the *y*-axis. A, mRNAs encapsidated in HIV-1 virus-like particles (VLPs); AC, mRNAs in cells producing HIV-1 VLPs; B, mRNAs encapsidated in murine leukemia virus (MLV) VLPs; BC, mRNAs in cells producing MLV VLPs.

As mentioned before, in the absence of the gRNA, HIV-1 Gag, as well as murine leukemia virus (MLV) Gag, packages cellular mRNAs without any obvious specificity [35]. However, although the concentration of the vast majority of the cellular mRNAs that are packaged in the absence of the gRNA is a reflection of their concentration in the cell, some mRNAs are preferentially packaged. One of these mRNA species is ankyrin repeat and SOCS box containing 1 (ASB-1) mRNA [35]. We noted that this mRNA has an unusually long 3′-UTR, and wondered whether this might contribute to the relatively efficient encapsidation of this mRNA. We have now explored this question by re-analyzing our earlier microarray data [35] on the mRNA species packaged in HIV-1 and MLV particles in the absence of Ψ-containing RNA. All data analyses were carried out using the R statistical programming language (R: A Language and Environment for Statistical Computing. R Core Team. Vienna Austria. https://www.R-project.org) and Bioconductor [38]. The raw data was normalized using Robust Multi-array Average (RMA) from the affy package [39]. As we did not have biological replicates, fold changes between the cellular and viral compartments were used for further analyses. The entire set of mRNAs was divided into bins according to the “fold change”, a measure of the selectivity of encapsidation of each species. Figure 2 shows the relationship between log_2_ (fold change) in each bin and the log_10_ (median 3′-UTR length) for the mRNAs in the bin. As can be seen, the mRNAs that are encapsidated with higher-than-average efficiency possess, on average, a significantly longer 3′-UTR than other mRNAs.

**Figure 3:**
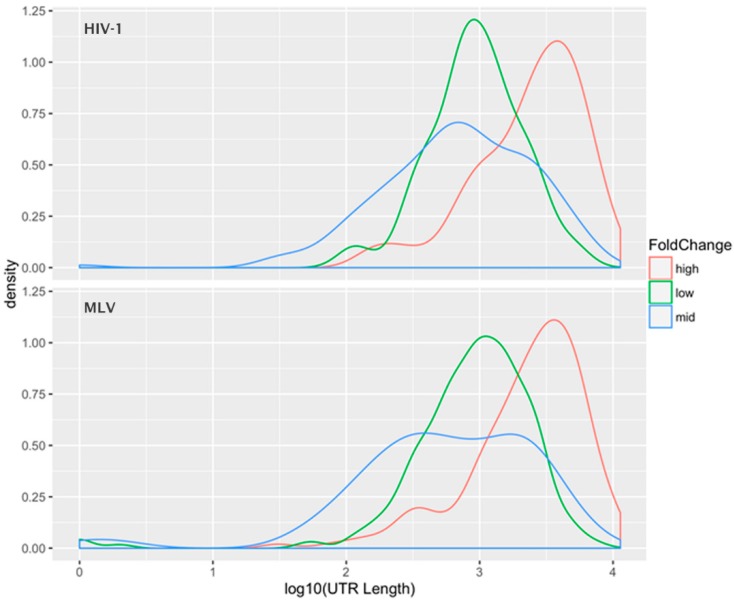
Preferential encapsidation of messenger RNA (mRNA) molecules with long 3′ UTRs. The log fold changes between messenger RNA (mRNA) measurements in the cellular and viral components were divided into groups of 1000 genes. The 1000 most excluded RNA species, labeled low; an “average” group representing the middle 1000 genes; and the 1000 most enriched mRNAs, the “high” group, are represented by different colored density plots. The plot depicts the log_10_ (UTR length) on the *x*-axis and the density on the *y*-axis. Upper panel: HIV-1; lower panel, MLV.

The data was also analyzed by selecting the 1000 mRNAs with the highest fold change, the 1000 species with the average fold-change, and the 1000 species with the lowest fold-change, i.e., those with the most preferential, average, and most excluded encapsidation efficiencies. Figure 3 shows the “density plot” (frequency distribution) of the 3′-UTR lengths for these three subsets of the mRNAs (for both HIV-1 and MLV). This analysis shows that the preferentially packaged mRNAs have unusually long 3′-UTRs, while there is little difference in the mean 3′-UTR lengths between the other two classes. A *t*-test between the UTR lengths in the “average fold-change” group and the “high fold-change” group was highly significant, with a *p*-value < 10^−15^. This relationship was observed for both HIV-1 and MLV Gag, suggesting that this apparent selectivity for long 3′-UTR RNAs is a general property of the retroviral assembly pathway, not unique to HIV-1 (a member of the lentivirus genus) or MLV (a gammaretrovirus). One possible explanation is that the 3′-UTR is a stretch of mRNA that is unoccupied by ribosomes; perhaps this provides sites to which Gag can bind in the absence of gRNA. Thus, RNAs with longer 3′-UTRs have a larger number of available Gag binding sites than RNAs with shorter 3′-UTRS. It is interesting to notice that the gRNA of retroviruses has an extremely long 3′-UTR.

In addition to the gRNA, wild-type viral particles contain host cell RNAs which can make up to one half of the total virion RNA mass. Most of these RNAs are tRNAs and non-coding RNAs and they are short (≤300 nts) compared to the length of the gRNA. Although it has been shown that, in the cytoplasm, the MA domain of Gag preferentially binds to tRNAs [14], there is evidence that packaging of specific lysine tRNAs depends on the incorporation of the lysyl-tRNA synthetase into HIV-1 virions (see, for example, [40]). This is significant because lysine tRNA_3_ is the primer for DNA synthesis during HIV-1 infection, and so this specific tRNA must be packaged in the particle. Moreover, it is a relatively low-abundance tRNA in the cell, and must therefore be packaged selectively. In turn, the synthetase is apparently packaged because it interacts with sequences in the CA domain of Gag [40]. A number of small cellular RNAs are packaged in the presence of the gRNA [41]; there are about 14 to 26 copies of 7SL RNA per HIV-1 virion (reviewed in [42]). Interestingly, while 7SL RNA is normally associated with signal recognition particle (SRP) proteins within the cell, these proteins do not seem to be packaged. This may mean that packaging, or at least binding, of these RNAs to Gag occurs immediately after they are transcribed [42].

## 7. Dimeric RNA as the Right Substrate

It is a well-known fact that in retroviruses the gRNA forms a stable dimer inside the mature virion [1,2]. From the perspective of RNA recombination, packaging two copies of the gRNA offers great advantages with respect to the viability of the virus progeny and viral evolution [1,2]. Moreover, many lines of evidence (summarized in [1,2]) indicate that RNA dimerization is required for selective packaging of the gRNA, as if the real packaging signal includes the three-dimensional structure of the dimeric form of the 5′ region of gRNA. This three-dimensional structure has been the focus of important recent research [43,44]. (In murine retroviruses, dimerization leads to a shift in register in some hairpins within Ψ. In turn, these shifts in register result in the exposure of short stretches of sequence that are base-paired in monomeric gRNA. The bases that are specifically unpaired in dimeric, but not in monomeric, gRNA are essential for selective packaging of the RNA, and it is clear that Gag binds to these specific bases [45,46]).

Transcripts representing HIV-1 gRNA can be dimerized in vitro in the absence of Gag and under very specific temperature and solution conditions. However, the rate of in vitro RNA dimerization can be increased by the presence of NC. A recent study in cell culture showed that it is very likely that RNA dimerization occurs close to the plasma membrane and that it requires the presence of Gag [47]. Gag, like its fragment NC, is a nucleic acid chaperone, capable of catalyzing the annealing of nucleic acids; it can promote RNA dimerization in vitro [48,49]. This study [47] showed that there is very little Gag associated with the gRNA before it is stably localized at the plasma membrane. This is consistent with immunoprecipitation assays of cytoplasmic Gag that show that the majority of RNA-associated Gag forms low-order aggregates (monomeric and dimeric) [20]. However, it is not clear if the assembly of these cytoplasmic Gag–RNA complexes is required for selective packaging of the gRNA. It should be noted that monomeric gRNA is an mRNA for Gag (and Gag–Pol, in which Gag is fused to other virus-coded proteins) in infected cells.

Nikolaitchik and co-workers showed that RNA dimerization is crucial for efficient RNA packaging and that HIV-1 regulates genome packaging by RNA copy number and not by RNA mass [50]. Another compelling result is the observation, originally reported by Sakuragi [51,52], that if an RNA molecule contains two dimerization signals, enabling it to form an “intramolecular dimer”, then this RNA can be efficiently packaged. This work shows that the dimeric structure of Ψ is the biologically relevant substrate required for selective packaging. In fact, Nikolaitchik et al. proposed that the dimeric Ψ promotes selective packaging of the gRNA by enhancing nucleation of virus assembly [50]. In other words, the structure of the dimeric Ψ lowers the energetic barrier for nucleation of virus assembly.

Although, for a long time, it was believed that the most important element within Ψ was stem-loop 3 (SL3), a recent study from Abd El-Wahab et al. showed that, in the presence of Gag, a bulge in SL1 (the stem-loop required for HIV-1 gRNA dimerization) is more protected from chemical and biochemical modifications than other regions of Ψ, including SL3 [32]. Abd El-Wahab and co-workers proposed that, in the context of Gag–Ψ interactions, the stem-loop required for dimerization of Ψ (SL1) is the most relevant element within Ψ. Their hypothesis, suggesting that Gag preferentially binds to the stem-loop required for gRNA dimerization, rather than to other elements within Ψ, is consistent with the hypothesis from Nikolaitchik and co-workers in which the dimeric Ψ is a nucleation site [50]. In fact, it seems plausible that selective packaging of the HIV-1 gRNA is achieved by Ψ-mediated enhancement of Gag–Gag interactions, such that binding of Gag to this RNA region allows for a more efficient nucleation of high-order Gag interactions.

## 8. Consequences of a Nucleation Mechanism

**Figure 4:**
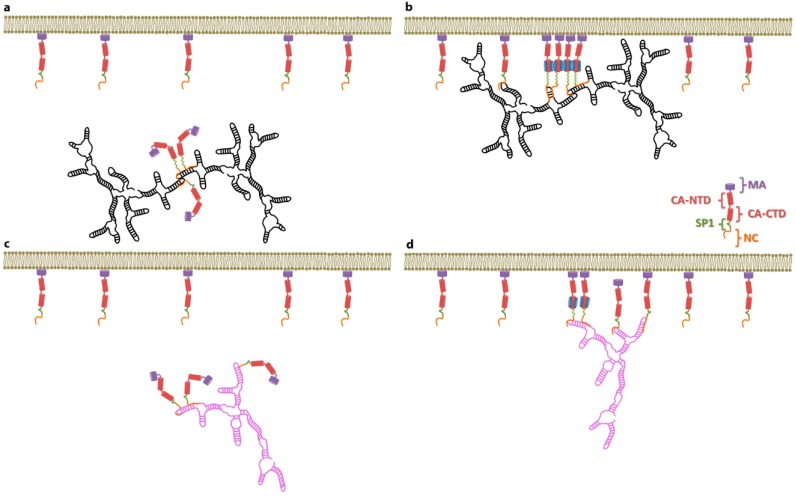
Schematic representation of the binding of HIV-1 Gag to the dimeric HIV-1 genomic RNA (gRNA) (**a**,**b**) and to non-Ψ RNA (**c**,**d**). (**a**) In the cytoplasm Gag binds to the HIV-1 gRNA by recognizing the RNA dimeric interface. Binding of Gag to Ψ increases the local concentration of Gag, thus promoting a conformational change in the SP1 domain from a random coil to an α-helix (green portion of Gag); (**b**) Once the HIV-1 Gag–dimeric gRNA complexes are bound to the plasma membrane, the specific Gag–RNA interactions promote a conformational change in Gag that enables high-order Gag–Gag interactions (blue interface); (**c**) In the absence of the HIV-1 gRNA Gag binds to mRNAs; however, because these interactions are non-specific, a higher Gag concentration is required to induce the conformational change in Gag; (**d**) When Gag–non-Ψ RNA complexes are bound to the plasma membrane, the local concentration of Gag is high enough to promote high-order Gag–Gag interactions. MA: matrix; CA-NTD: N-terminal domain of the capsid; CA-CTD: C-terminal domain of the capsid; SP1: spacer 1; NC: nucleocapsid.

Earlier in this review, we described our findings on the SP1 domain of Gag, which led us to suggest that a conformational change in SP1 switches Gag into an assembly-ready state [23]. In some biological systems the nucleation of high-order protein complexes (e.g., immature virions) is driven by protein-protein interactions. Initiation of this process has to overcome an energetic barrier (activation energy); hence a nucleation process can be accelerated if the activation energy is lowered. This can be achieved by the presence of a nucleation site or a “seed”. In the simplest scenario, a nucleation site acts by enhancing protein-protein interactions such that the energy gained by protein multimerization overcomes the loss of entropy due to the decrease of free protein, and therefore lowers the activation energy. Based on the idea proposed by Nikolaitchick and co-workers [50], on some of the data discussed here, and on these physical principles, we can ask the following question; is selective packaging of the HIV-1 gRNA driven by Ψ acting as a nucleation site for virion assembly? A hypothesis in which selective packaging of the HIV-1 gRNA is achieved by a Ψ lowering the energy needed to nucleate virion assembly is consistent with the fact that HIV-1 VLP assembly can be efficiently supported by cellular mRNAs, but that this assembly process is completely inhibited in the presence of the gRNA. In the context of this nucleation scenario it is possible that the dimeric Ψ acts as a “seed” such that binding of just a few Gag molecules to this substrate is enough to activate the SP1 conformational switch, exposing new Gag–Gag interfaces and therefore nucleating efficient virion assembly (see Figure 4). According to this hypothesis, VLP assembly can occur in the absence of the dimeric Ψ, but this process has a higher activation energy because binding to the non-Ψ is less efficient in triggering the exposure of the interfaces in Gag that are required for efficient Gag multimerization (nucleation).

In order to determine the validity of this hypothesis one would need to be able to measure Gag–Gag interactions in the presence and absence of RNA (the substrate that acts as an assembly catalyst). More importantly, one would have to determine if the strength of Gag–Gag interactions or the conformation of Gag in Gag–RNA complexes depends on the sequence of the bound RNA (i.e., Ψ versus non-Ψ RNAs, as well as monomeric versus dimeric Ψ). This hypothesis could be tested by measuring the rate of VLP assembly, or virus budding, in the presence of Ψ^+^ and Ψ^−^ RNAs, both in vitro and in cell culture. Testing this hypothesis should enable us to understand how the strength of Gag–Gag interactions controls assembly, both in vitro and in cell culture, and how the modulation of these interactions leads to selective packaging of the gRNA. Furthermore, the existence of a Ψ-dependent nucleation complex might suggest new therapeutic strategies that would inhibit packaging of the gRNA.
